# Treating Cancer as an Infectious Disease—Viral Antigens as Novel Targets for Treatment and Potential Prevention of Tumors of Viral Etiology

**DOI:** 10.1371/journal.pone.0001114

**Published:** 2007-10-31

**Authors:** Xing Guo Wang, Ekaterina Revskaya, Ruth A. Bryan, Howard D. Strickler, Robert D. Burk, Arturo Casadevall, Ekaterina Dadachova

**Affiliations:** 1 Department of Nuclear Medicine, Albert Einstein College of Medicine, Bronx, New York, United States of America; 2 Department of Epidemiology and Population Health, Albert Einstein College of Medicine, Bronx, New York, United States of America; 3 Department of Pediatrics, Albert Einstein College of Medicine, Bronx, New York, United States of America; 4 Department of Microbiology and Immunology, Albert Einstein College of Medicine, Bronx, New York, United States of America; 5 Department of Obstetrics and Gynecology and Women's Health, Albert Einstein College of Medicine, Bronx, New York, United States of America; 6 Department of Medicine, Albert Einstein College of Medicine, Bronx, New York, United States of America; Ordway Research Institute, United States of America

## Abstract

**Background:**

Nearly 20% of human cancers worldwide have an infectious etiology with the most prominent examples being hepatitis B and C virus-associated hepatocellular carcinoma and human papilloma virus-associated cervical cancer. There is an urgent need to find new approaches to treatment and prevention of virus-associated cancers.

**Methodology/Principal Findings:**

Viral antigens have not been previously considered as targets for treatment or prevention of virus-associated cancers. We hypothesized that it was possible to treat experimental HPV16-associated cervical cancer (CC) and Hepatitis B-associated hepatocellular carcinoma (HCC) by targeting viral antigens expressed on cancer cells with radiolabeled antibodies to viral antigens. Treatment of experimental CC and HCC tumors with ^188^Re-labeled mAbs to E6 and HBx viral proteins, respectively, resulted in significant and dose-dependent retardation of tumor growth in comparison with untreated mice or mice treated with unlabeled antibodies.

**Conclusions/Significance:**

This strategy is fundamentally different from the prior uses of radioimmunotherapy in oncology, which targeted tumor-associated human antigens and promises increased specificity and minimal toxicity of treatment. It also raises an exciting possibility to prevent virus-associated cancers in chronically infected patients by eliminating cells infected with oncogenic viruses before they transform into cancer.

## Introduction

It has been estimated that nearly 20% of human cancers worldwide have an infectious etiology [1]. Most of these tumors are of viral origin, and include firmly established associations of hepatitis B virus (HBV) and hepatitis C virus (HCV) with hepatocellular carcinoma; and of human papillomavirus (HPV)-with cancers of the cervix, anus, vulva, vagina; as well as associations of oropharynx Epstein-Barr virus (EBV) with lymphoma and nasopharyngeal carcinoma; human T lymphotropic virus type 1 (HTLV-1)-with adult T-cell leukemia/lymphoma, and human herpes virus 8 (HHV-8)-with Kaposi sarcoma [2]–[7]. In combination, these virus-associated tumors represent a burden of approximately 1.3 million cases of cancer each year, with HBV/HCV-associated liver cancer accounting for 523,000 cases, and HPV-associated tumors accounting for 561,000 cases [8]. The need to find new approaches to the treatment and prevention of virus-associated cancers is obvious and urgent.

Radioimmunotherapy (RIT) utilizes antigen-antibody binding to deliver cytotoxic doses of particulate radiation to tumor cells [9], [10]. RIT, for example, has been used to successfully treat refractory and recurrent lymphomas, with two radiolabeled monoclonal antibodies (mAb) targeted against CD20 (Zevalin® and Bexxar®) having received FDA approval for this purpose. It is likely, in fact, that RIT will become a first line treatment for follicular lymphoma [11]. This “traditional” cancer RIT targets “self” antigens. Recently, we demonstrated that RIT has also broad potential for the treatment of fungal and bacterial infections through targeting of microbial antigens with radiolabeled mAbs in experimental models of fungal and bacterial infections [12], [reviewed in 13]. In addition, we found that HIV-1 infected cells could be eliminated in vitro and in vivo by targeting gp120 and gp41 viral glycoproteins expressed on the surface of infected cells with radiolabeled viral protein-specific mAbs [14].

We hypothesized that RIT targeted against viral antigens could be used in the treatment of a broad range of viral infectious diseases and virus-associated tumors [15], [16]. Many virus-associated cancers express viral antigens either internally or on their surfaces. It is important to note that even viral antigens expressed intracellularly are potential targets for RIT, since tumor cell turnover is likely to result in the release of these proteins into the interstitial space of the tumor. This approach is fundamentally different from the previously described uses of RIT which target tumor-associated antigens that are “self” (i.e., human) proteins. By targeting viral and not “self” proteins, it is hoped that radiolabeled mAbs can be more specifically concentrated within tumor tissue, resulting in greater efficacy and less toxicity. Here we describe the proof-of-principle experiments aimed at demonstrating the feasibility of treating experimental HPV16-associated cervical cancer (CC) and Hepatitis B-associated hepatocellular carcinoma (HCC) by targeting viral antigens expressed on cancer cells with radiolabeled antibodies to viral antigens.

## Results

### Selection of a cell line-antigen combination to act as an experimental cervical cancer (CC) model

**Figure 1:**
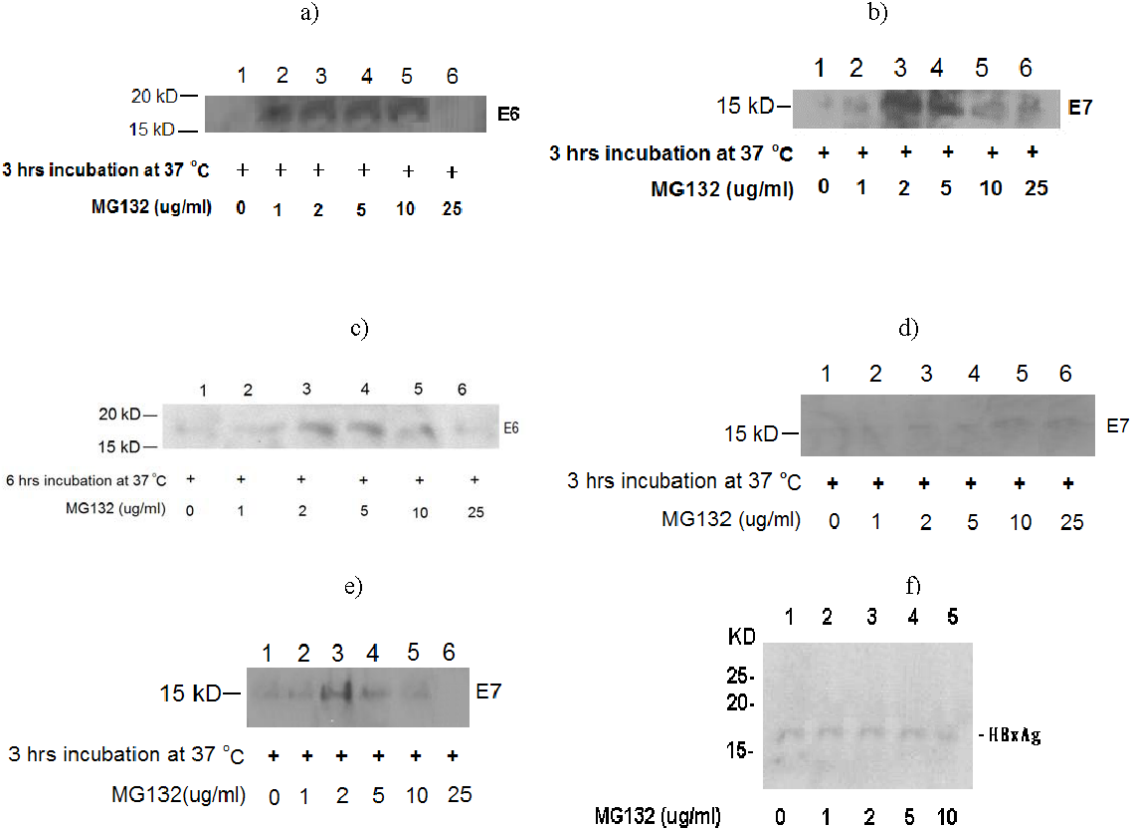
Expression of E6 and E7 in human cervical carcinoma cell lines and of HBx in hepatocellular carcinoma cell line by Western blot and effect of MG132 proteasome inhibitor treatment on the levels of E6, E7 and HBx in these cell lines: a) E6 from protein extracts of CasKi cells treated with MG132 for 3 hrs; b) E7 from protein extracts of CasKi cells treated with MG132 for 3 hrs; c) E6 from protein extracts of CasKi cells treated with MG132 for 6 hrs; d) E7 from protein extracts of SiHa cells treated with MG132 for 3 hrs; e) E7 from protein extracts of HeLa S3 cells treated with MG132 for 3 hrs; f) HBX from protein extracts of Hep 3B2.1-7 cells treated with MG132 for 3 hrs.

To evaluate the potential of RIT to target viral antigens in cancers of viral etiology, we needed to identify tumor cell lines that expressed the target antigen and could also be implanted into nude mice. We selected HPV16 and HPV18 cell lines, since these two HPV types account for approximately 70% of cervical cancers and a significant fraction of head and neck tumors [17], [18]. The E6 and E7 oncoproteins were considered the best potential antigenic targets, since these proteins are expressed in essentially all cervical cancer cells, whereas other viral genes may be lost. Mutational analysis has shown that the E6 and E7 viral oncoproteins are necessary and sufficient for the immortalization of human cells by HPV. Therefore, we assessed by Western blot the expression of E6 and E7 in three human cervical carcinoma cell lines–HPV16-positive CasKi and SiHa cell lines and HPV18-positive HeLa S3 cell line. While CasKi cells expressed both E6 and E7 antigens (Fig. 1a, b), SiHa and HeLa S3 cell lines had no measurable expression of E6 antigen (results not shown) but did express E7 protein (Fig. 1d, e); albeit, the level of E7 expression was low in SiHa cells (Fig. 1d).

**Figure 2:**
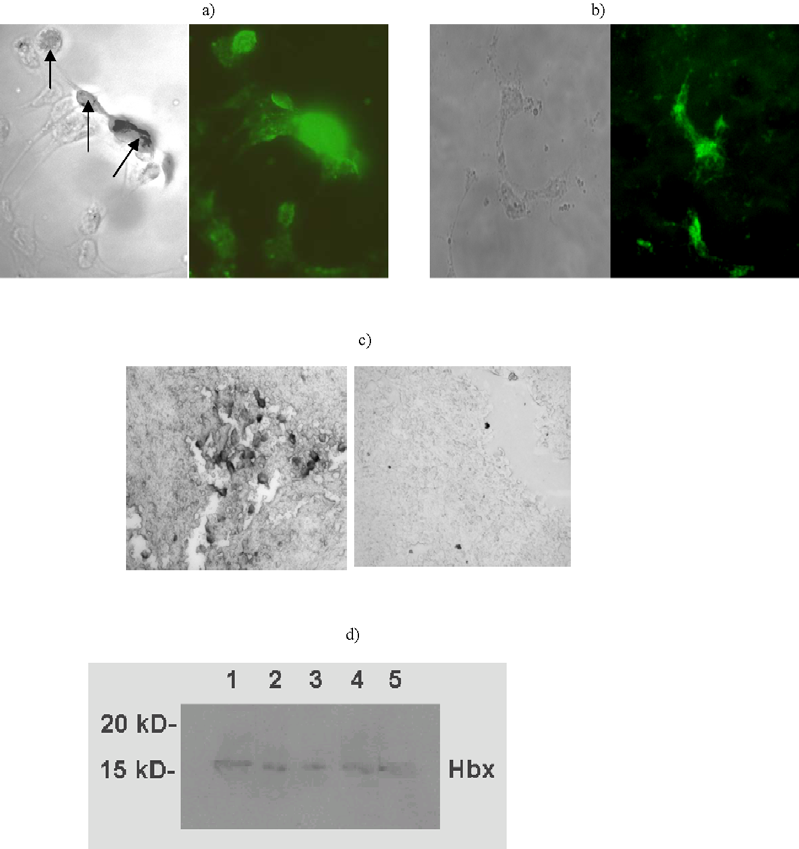
Detection of viral antigens in tumor cells and in tumors: a, b) immunofluorescence of fixed and permeabilized tumor cells. Left panels show light microscopy images of the cells. Heavily damaged cells are marked with arrows. Right panels show immunofluorescent images of the same slides treated with viral protein-specific mAbs followed by FTIC-conjugated polyclonal antibody to mouse IgGs: a–CasKi cells and E6-specific C1P5 mAb, b-Hep 3B2.1-7 cells and HBx-specific 4H9 mAb; c) immunohistochemistry of CasKi tumors. Left panel shows binding of E6-specific mAb C1P5. Right panel shows absence of binding of control mAb 18B7; d) western blot of Hep 3B2.1-7 tumor with HBx-specific mAb 4H9.

The proteasome inhibitor MG132 reduces the degradation of ubiquitin-conjugated proteins in mammalian cells without affecting ATPase or isopeptidase activities. MG132 has been reported to result in increased levels of E6 and E7 proteins in cervical cancer cells [19], [20]. As higher amounts of target antigens can potentially improve RIT results, we investigated the influence of pre-treatment of CC cells with proteasome inhibitor MG132 on the levels of E6 and E7 expression. Fig. 1a and b shows that in CasKi cells pre-treatment with MG132 caused an increase in expression of both E6 and E7, with the greatest level of expression achieved with use of 2 and 5 µg/mL of MG132 which subsided when higher doses were used. Prolongation of the incubation period of CasKi cells with MG132 did not result in greater E6 expression. In fact, we observed decreased E6 expression with prolonged CasKi cell exposure to MG132 (Fig. 1c), which might reflect an increase of protein degradation after more than 3 hr of incubation. Similar experiments were performed for E7 protein in SiHa and HeLa S3 cell lines (Fig. 1d,e). SiHa cells did not demonstrate an appreciable increase in the E7 levels (Fig. 2d); whereas, E7 levels did increase slightly for HeLa S3 cells treated with 1 and 2 µg/mL MG132 (Fig. 1e). Given reliable and high expression of E6 in CasKi cells as well as their ability to produce tumors in nude mice-we selected CasKi cells and E6 protein for further in vitro and in vivo experiments.

### Selection of a cell line-antigen combination to act as an experimental Hepatitis B-associated hepatocellular carcinoma (HCC) model

We evaluated two Hepatitis B-associated viral proteins as potential targets for RIT-HBx and PreS2. HBx is suspected to have a role in hepatocarcinogenesis and, unlike other potential choices, HBx has no homology to host proteins. Further, the gene that codes for HBx is retained even when the HBV genome becomes integrated in HCC, whereas other HBV genes may be lost [21], [22]. PreS2 is also suspected to have a role in hepatocarcinogenesis (i.e., through transactivation of cellular genes important in growth control) [22]. HBx protein was consistently detected by Western blot of HCC cell line Hep 3B2.1-7 using 4H9 mAb, and its expression was independent of pre-treatment with MG132 proteasome inhibitor (Fig. 1f), whereas preS2 was not detected (results not shown). Therefore, we selected the Hep 3B2.1-7 cell line and HBx protein combination for further experiments.

### Binding of antibodies to viral antigens in non-viable cells

To find out if antibodies to viral proteins which were identified as targets for RIT will be able to bind to viral proteins in non-viable tumor cells, we performed immunofluorescence of fixed and permeabilized CasKi and Hep 3B2.1-7 cells with mAbs C1P5 to E6 and 4H9 to HBx proteins, respectively, followed by FITC-conjugated polyclonal antibody to mouse IgG. While the binding of C1P5 mAb to the cells which were almost intact was weak, the heavily damaged cells with penetrable membranes showed bright fluorescence pointing to binding of C1P5 to E6 (Fig. 2a). The fixation and permeabilization also made possible for mAb 4H9 to bind to HBx protein (Fig. 2b). No binding of control IgG1 mAb to fixed and permeabilized CasKi or Hep 3B2. 1-7 cells was observed (results not shown).

### Expression of viral proteins in the tumors

To confirm that CasKi and Hep 3B2.1-7 cells continued to express E6 and HBx viral antigens, respectively, in the tumors induced in nude mice, we performed immunohistochemistry and immunoblot for E6 and HBx, respectively. Western blot was chosen for Hep 3B2.1-7-induced tumors based on the literature reporting difficulties in reliably detecting HBx protein in organs by immunohistochemistry [23]. There was intense staining of E6 with specific mAb C1P5 in CasKi tumors (Fig. 2c, left panel) with no staining observed with control IgG1 mAb (Fig. 2c, right panel). Western blot of Hep 3B2.1-7-induced tumors revealed the presence of HBx protein (Fig. 2d). Thus, the presence of the target viral proteins in CC and HCC experimental tumors provided the possibility of targeting these antigens in vivo with radiolabeled mAbs for scintigraphic imaging and therapy.

### Biodistribution of radiolabeled mAbs to viral proteins in CasKi and Hep 3B2.1-7-tumor bearing nude mice

**Figure 3:**
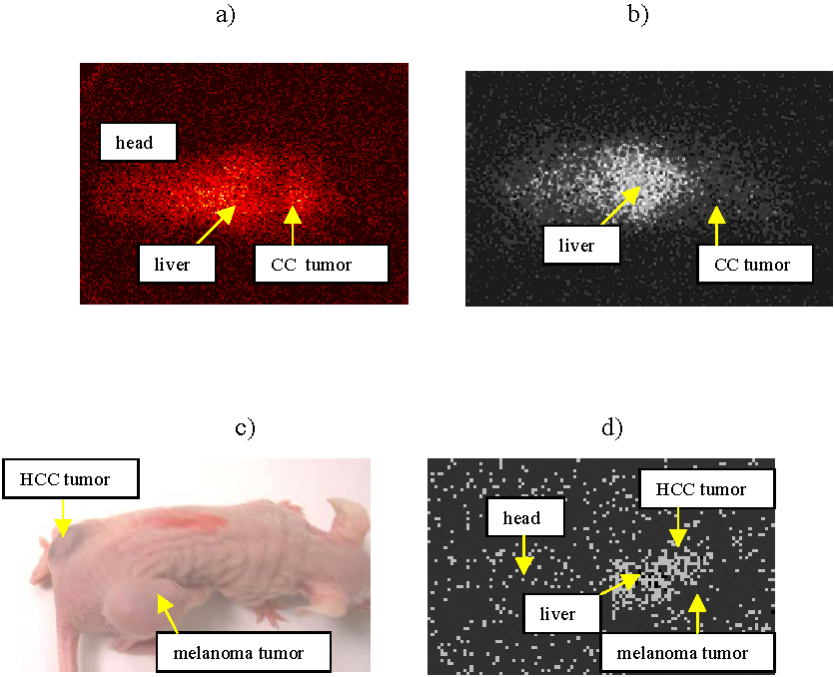
Scintigraphic images of tumor-bearing mice 24 hours post-injection with: a) CasKi tumor, E6-specific mAb ^188^Re-C1P5 mAb; b) CasKi tumor, control ^188^Re-18B7 mAb; c) Hep 3B2.1-7 and A2058 human metastatic melanoma tumors, HBx-specific ^188^Re-4H9 mAb.

We performed imaging and biodistribution experiments with ^188^Re-radiolabeled C1P5 and 4H9 mAbs in CasKi and Hep 3B2.1-7-tumor bearing nude mice, respectively, to ascertain the localization of mAbs to the tumors. At 24 hr post-injection the CasKi tumor was visible on the scintigraphic image of a mouse injected with ^188^Re-C1P5 mAb (Fig. 3a) as opposed to the image of a mouse injected with irrelevant ^188^Re-18B7 mAb (Fig. 3b). We also calculated the tumor to muscle ratio from the 48 hr biodistribution results which was 10∶1 for ^188^Re-C1P5 versus 3∶1 for ^188^Re-18B7. The overall uptake of ^188^Re-C1P5 mAb in the tumors at 48 hr post-injection was 2.0(±0.3)% of the injected dose per gram tumor. Other organs like liver, spleen and blood showed the levels of uptake characteristic of IgG1 mAbs. For Hep 3B2.1-7 we utilized another approach to prove the specificity of mAb uptake in the tumor by using a model when a mouse carries two different tumors–one the tumor of interest and another–irrelevant control tumor. Nude mice carried A2058 human metastatic melanoma tumor on the right flank and Hep 3B2.1-7 on the left (Fig. 3c). The mice were injected with ^188^Re-4H9 mAb and imaged scintigraphically at 24 hr post-injection. The antibody localized to the Hep 3B2.1-7 tumor as opposed to the irrelevant control melanoma tumor (Fig. 3d). The ability of radiolabeled mAbs to viral antigens to localize to these tumors in mice justified an assessment of RIT in these in vivo cancer models.

### RIT of CasKi and Hep 3B2.1-7-tumor bearing nude mice

**Figure 4:**
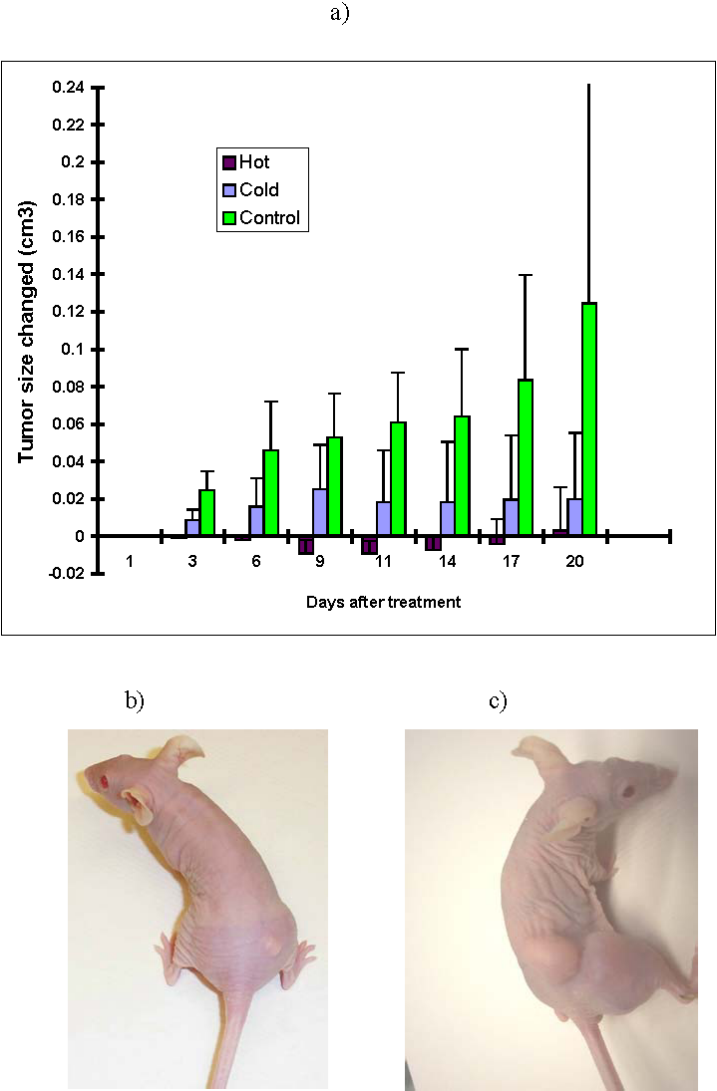
Radioimmunotherapy of CasKi tumors in nude mice: a) changes in tumor volume; b) mouse treated with 350 µCi ^188^Re-C1P5 mAb; c) control mouse (both mice shown on Day 20 post-treatment).

To evaluate the therapeutic effect of ^188^Re-C1P5 mAb in CasKi tumor-bearing mice, the animals were injected with either 350 µCi ^188^Re-C1P5 mAb, or matching amounts (30 µg per mouse) of unlabeled (“cold”) C1P5 mAb or left untreated. Fig. 4a shows the change in tumor volume in the treated and control groups. RIT with 350 µCi ^188^Re-C1P5 mAb completely arrested tumor growth and resulted in its volume reduction (Fig. 4a and b), while untreated tumors grew aggressively (Fig. 4a and c) and untreated control mice had to be sacrificed on Day 20 post-treatment (P<<0.01). Interestingly, administration of “cold” C1P5 mAb also resulted in significant retardation of the tumor growth, which can be due to the induction of inflammation and complement cascades by the mAb.

**Figure 5:**
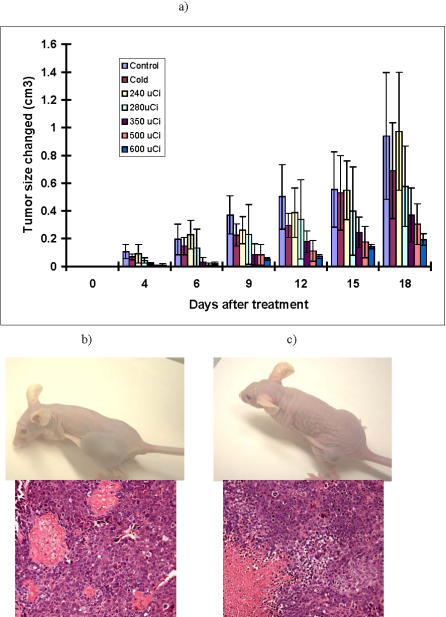
Radioimmunotherapy of Hep 3B2.1-7 tumors in nude mice: a) changes in tumor volume; b) control untreated mouse; c) mouse treated with 600 µCi ^188^Re-4H9 mAb. Both mice shown on Day 18 post-treatment. In b and c lower panels show H&E stained tumors at the completion of the experiment.

For RIT of Hep 3B2.1-7 tumors in nude mice we initially employed the same experimental design as for CasKi tumors by using 350 µCi ^188^Re-4H9 mAb, “cold” mAb-treated controls and untreated groups. Administration of 350 µCi ^188^Re-4H9 mAb resulted in slower tumor growth but did not arrest it like in case of CasKi tumors. “Cold” 4H9 mAb did not have an effect on the tumor growth. To find the most efficient dose of radiolabeled mAb for retarding the growth of very aggressive Hep 3B2.1-7 a dose response experiment was done. The therapeutic effect of ^188^Re-4H9 mAb began to manifest itself at a dose of 280 µCi and the effect increased with every subsequent increase in the dose (Fig. 5a) (P<0.02). At the completion of the experiment the tumors from the control untreated mice and mice in the highest 600 µCi group were analyzed histologically. The control tumor consisted of moderately differentiated hepatoid cells with scattered small foci of necrosis, fibrin thrombosis, and hemorrhage. Neoplastic cells had ample eosinophilic to finely vacuolated cytoplasm and medium-sized nuclei to very large and anaplastic nuclei containing multiple large eosinophilic nucleoli with multinucleated cells occasionally evident. The mitotic index was high and there were scattered apoptotic cells (Fig. 5b). In contrast, the RIT-treated tumors had significantly more necrosis and hemorrhage than seen in the controls. The morphologic appearance of the tumor cells often had a more vacuolated cytoplasm suggesting degeneration (Fig. 5c).

## Discussion

We performed proof-of-principle in vitro and in vivo experiments to establish the feasibility of targeting viral antigens in tumors of viral etiology with radiolabeled antibodies for therapy. To this end, we studied experimental cervical carcinoma (CC) and hepatocellular carcinoma (HCC) models, since these cancers are etiologically related to HPV and HBV/HCV, respectively, and have major public health implications world-wide.

The first challenge was the choice of target viral antigens in CC and HCC. In HPV-associated CC we identified E6 and E7 oncoproteins as potential targets for RIT, because they are thought to be expressed in all cervical cancer cells; whereas, other viral genes may be lost during the multi-stage process of tumorigenesis. Similarly, in HCC, HBx expression is thought to be maintained. There is, however, an important concern in targeting these three proteins with radiolabeled mAbs–their subcellular location. Both E6 and E7 are located within the nucleus, and HBx is found in the nucleus and occasionally in the cytoplasm. As a consequence, the radiolabeled mAbs to these proteins can bind only to their respective antigens when they are released from the dead cells or when tumor cells are permeabilized. To conduct these experiments, we chose the CasKi cell line as a CC model, since we found it expressed E6 and E7 at high levels in vitro and the tumors grew aggressively in nude mice after the latent period. The Hep 3B2.1-7 cell line was chosen as a HCC model, after we observed that it expressed HBx at high levels and displayed fast tumor growth in mice.

The immunofluorescence of the tumor cells and immunohistochemistry and Western blot of the tumors revealed ample amounts of E6 and HBx antigenic targets in CasKi- and Hep 3B2.1-7-induced tumors, respectively (Fig. 2). The presence of accessible antigen for the radiolabeled mAbs is probably a result of protein release from tumor cells undergoing rapid turnover. Consequently, the radiolabeled antibodies accumulated in the tumor tissue such that they could be imaged scintigraphically (Fig. 3). One of the advantages of targeting viral antigens in the tumors that they are only found in malignant cells. In contrast, Chen and colleagues observed no differences in the tumor uptake for both specific and non-specific radiolabeled antibodies in their biodistribution experiments when they targeted intranuclear histones [24].

The ability of radiolabeled mAbs against viral proteins to deliver cytotoxic radionuclide 188-Rhenium to the tumor cells facilitated the successful outcomes of RIT with these mAbs in tumor-bearing mice. The administration of 350 µCi dose of E6-binding ^188^Re-C1P5 mAb to CasKi tumor-bearing mice completely stopped tumor growth and even resulted in its regression (Fig. 4). It is also likely that there was some added therapeutic benefit from the antibody itself as unlabeled antibodies can mediate inflammatory reactions and activate compliment. In the future, it will be worthwhile to investigate whether increased cellular levels of E6 and E7 oncoproteins could be obtained with pretreatment use of MG132 in vivo, and whether this results in a therapeutic benefit. The dose response experiments in mice bearing Hep 3B2.1-7 HCC-tumors clearly demonstrated a dose dependence for the therapeutic effect (Fig. 5). The fact that higher doses of radiolabeled mAb were needed to produce a therapeutic effect in HCC than in cervical tumor might reflect the aggressiveness of Hep 3B2.1-7 and the known radioresistance of liver tumors in comparison to the relatively radiosensitive cervical carcinomas [25]. It is also noteworthy that therapeutic gains in both experimental tumors were achieved with doses of radioactivity that are well below the maximum tolerated dose of approximately 800 µCi for ^188^Re-labeled IgG1s in mice [26], and as such the doses used were not expected to produce any short- or long-term toxicities.

It is important to emphasize that when treating viral-associated cancers by targeting viral antigen not every cell in the tumor needs to express viral antigens for a therapeutic effect. Long range emitters such as ^188^Re (emission range in tissue 10 mm) emit radiation in a 360° sphere and consequently can kill cells in the vicinity of the antigen location. Furthermore, a high concentration of the targeted viral antigen is probably not required for the delivery of a therapeutic dose to the tumor, according to our recent model of RIT for melanoma (targeted against melanin which is also an intracellular antigen). This model showed that the radiation dose delivered to the tumor is largely independent of melanin (antigen) concentration [27].

This approach also holds the promise to prevent viral-associated cancers in chronically infected individuals-for example, persistence of HPV infection in HIV-infected individuals puts them at significant risk of developing HPV-associated cancers. While there is immunotherapy for HBV and HCV, many patients do not achieve viral clearance and the treatment is associated with substantial morbidity. There is also no vaccine for HCV, and many millions of people worldwide are infected with HBV despite the availability of an effective vaccine. Cells persistently infected with HPV, HBV, HCV or other viruses could potentially be eliminated with RIT targeted to viral antigens before they transform into malignant phenotype.

In conclusion, we performed in vitro and in vivo experiments to assess a novel strategy to treat virus associated tumors using radiolabeled mAbs targeted against viral proteins. This strategy is fundamentally different from prior uses of RIT in oncology that target tumor-associated human antigens thus resulting in significant uptake of radioactive antibody in normal tissues, leading to toxicity. The results of our study suggest that by targeting instead viral and not self-proteins it may be possible for radiolabeled mAbs to concentrate more specifically within tumor tissue, resulting in greater efficacy and less toxicity. This strategy also raises an exciting additional possibility to prevent virus-associated cancers in chronically infected patients by eliminating cells infected with oncogenic viruses before they transform into cancer.

## Methods

### Antibodies

Mouse mAbs from Abcam C1P5 to HPV16 E6+HPV18 E6 and TVG 701Y to HPV16 E7 were used in CC experiments. Mouse mAb 4H9 to HBV HBx (Aviva, Cat# AVAMM2005) and mouse mAb S26 to HBV protein Hbs (Gene Tex Inc. Cat# GTX18797) which cross-reacts with HBsAg preS2 antigen were used in HCC experiments. Murine 18B7 mAb (IgG1) to *C. neoformans* [28] was used as an irrelevant control for all specific antibodies. Rabbit polyclonal antibody conjugated with alkaline phosphatase to mouse IgG H&L was purchased from Abcam and used as secondary antibody in Western blot.

### Cell lines and cell cultivation

Three human cervical carcinoma cell lines: Hela S3, SiHa and CasKi were purchased from ATCC (Manassas, VA). Cells were grown routinely in DMEM/HAM F-12K (Sigma, 1∶1) containing 10% FBS (Sigma) and 1% Penicillin-streptomycin solution (Sigma, penicillin 10,000 U and streptomycin 10 mg/ml) at 37°C in 5% CO_2_ incubator. The cell line Hep 3B2.1-7 (*Homo sapiens* hepatatocellular carcinoma) was purchased from ATCC. It contains an integrated hepatitis B virus genome and was derived from the hepatocellular carcinoma tissue of 8-year old black boy. Cells were grown routinely in Eagle's Minimum Essential Medium (EMEM) (ATCC) containing 10% FBS (Sigma) at 37°C in 5% CO_2_ incubator. Cell layers in 75 ml flask was dispersed by adding 2 ml 1× Trypsin-EDTA solution (Sigma, 0.25% (w/v) trypsin−0.53 mM EDTA) at room temperature for 15 minutes. A2058 cell line derived from a lymph node metastasis from patient with malignant melanoma was obtained from ATCC. The cells were maintained as monolayers in Dulbecco's Modified Eagle's Medium with 4 mM L-Glutamine, 4.5 g/L glucose, 1.5 g/L sodium bicarbonate, supplemented with 10% fetal bovine serum and 5% penicillin-streptomycin solution at 37°C and 5% CO_2_, and harvested by using 0.25% (w/v) Trypsin-EDTA solution. Routine maintenance of all cell lines was performed according to the ATCC protocols.

### Western blots

Cell pellets were suspended in the lysis buffer (4% SDS, 20% glycerol, 0.5 M TrisHCl (pH 6.8), 0.002% bromophenol blue and 10% β−mercaptoethanol). Protein samples were boiled in water for 15 min before running SDS-PAGE. Twenty five-thirty µl protein solution was loaded into each well of 12% precast SDS-PAGE gel (Bio-Rad). SDS-PAGE was used to monitor the relative protein amounts in the samples. Twelve percent SDS-PAGE gel was used to separate proteins, and electrophoresis was performed using Mini-Protean® 3 Cell system (Bio-Rad). After electrophoresis, the gel was transferred into the PVDF transfer buffer (25 mM Tris, 190 mM glycine and 2.5% (v/v) methanol) for 5 min. Then, proteins were transferred from the gel to the Immun-Blot™ PVDF membrane (Bio-Rad) on Semi-dry Electrophoretic Transfer Cell (Bio-Rad) at 15 V for 17 min. The membrane was soaked in the blocking buffer (25 mM TrisHCl (pH7.6), 1 mM EDTA and 150 mM NaCl) for 5 min, and then transferred into the blocking solution (5% non-fat dry milk in the blocking buffer), shaking gently for an hour. The membrane was incubated in TBST solution (0.1% Tween-20, 25 mM TrisHCl (pH7.6) and 500 mM NaCl) containing 1∶3000 diluted primary antibody at room temperature for 1 hour with gentle shaking. Then, the membrane was washed with TBST three times (10 min per wash). The membrane was hybridized by the secondary antibody conjugated with alkaline phosphatase (Rabbit polyclonal to mouse IgG H&L (alkaline phosphatase)) in TBST with a 1∶100,000 dilution. After three washes with TBST, the membrane was incubated in CDP-Star™ chemiluminescent substrate solution (Sigma) for 5 min, and then exposed to CL-XPosure™ film (Pierce). The film was developed as per manufacturer's instructions.

### MG132 treatment of cells

MG132 (Z-LLL-CHO, MW = 457.6) purchased from CALBIOCHEM is a potent, reversible and cell-permeable proteasome inhibitor (Ki = 4 nM). MG132 was first dissolved in a drop of 100% ethanol followed by DMEM/HAM F-12K medium without addition of FBS, and the stock solution was stored at 4°C. Cell culture was harvested and transferred into 6 sterile test tubes. Each tube contained 0.5–1 ml cell culture (cell concentration was ∼10^6^ cells/ml), and cells were allowed to grow at 37°C in 5% CO_2_ incubator overnight. Then, MG132 solution was added to the tubes for the final concentrations of 0, 1, 2, 5, 10 or 25 µg/ml (0, 2.1, 4.2, 10.5, 21 or 52 µM, respectively). The cells were incubated under the same conditions for another 3 or 6 hours. Finally, cells were harvested by centrifugation and clarified by washing with PBS.

### Radioisotope and radiolabeling of the antibodies

The beta-emitter ^188^Re with a half life of 16.9 hours was eluted in the form of sodium perrhenate Na^188^ReO_4_ from an ^188^W/^188^Re generator (Oak Ridge National Laboratory, Oak Ridge, TN). Antibodies were labeled with ^188^Re directly through binding of reduced ^188^Re to the generated−SH group on the antibodies as previously described [11].

### Immunofluorescent detection of viral antigens in tumor cells

The tumor cells were grown in the slide chambers at 37°C for 12 hrs. The medium was removed and the cells were washed with PBS three times. Throughout the procedure, the cells were washed 3 times with PBS after each treatment. They were fixed with 4% paraformaldehyde at room temperature for 20 min followed by permeabilization with 0.3% Triton-X100 in PBS at room temperature for 10 min. Fixed and permeabilized cells were blocked with 5% BSA plus 0.1% Triton X-100 in PBS at room temperature for 30 min. Viral antigens-specific mAbs were diluted 1∶200 with the above BSA and Triton X-100 solution and incubated with the cells at room temperature for 60 min. FTIC-conjugated rabbit polyclonal antibody to mouse IgG in 1∶300 dilution was added to the cells for 60 min incubation at room temperature in the dark. The slides were viewed with an Olympus AX70 microscope (Melville, NY) equipped with a FITC filter.

### Tumor models

All animal studies were carried out in accordance with the guidelines of the Institute for Animal Studies at the Albert Einstein College of Medicine. Human cervical carcinoma CasKi and human hepatocellular carcinoma Hep 3B2.1-7 cell lines were chosen as the tumor models based on its expression of E6 and HBx, respectively, and their tumorigenecity in nude mice. Six-week-old female Nu/Nu CD1 nude mice purchased from Charles River were injected into the right flank with 10^7^ CaSki or Hep 3B2.1-7 cells in 0.1 ml DMEM medium containing 10% fetal calf serum. CasKi tumors began to appear around 60 days after injection, Hep 3B2.1-7 tumors-10 days post cell injection. For biodistribution of HBx-specific mAb, nude mice were inoculated with 10^7^ A2058 human metastatic melanoma cells and 10^7^ Hep 3B2.1-7 cells into the right and left flanks, respectively. Biodistribution and therapy experiments were initiated when the tumors reached 0.3–0.7 cm in diameter.

### Immunohistochemical detection of HPV E6 protein in CasKi tumors

The tumors taken from CasKi tumor-bearing mice were fixed in 10% buffered formalin overnight, followed by placing in 70% ethanol. The paraffin-embedded tumor tissues were cut into 5 µm slides. The sections were deparaffinized in xylene and dehydrated in graded ethanol continuously, and pre-treated with citrate buffer (pH 6.0) at 100°C for 20 min to retrieve the antigen. The sections were treated with 3% hydrogen peroxide in methanol in order to block the endogenous peroxidase activity. IHC staining of the tumor tissue sections was then performed using Histostain®-Plus Kits Zymed® 2^nd^ Generation LAB-SA Detection System (Invitrogen) according to the manufacturer's instruction. Mouse mAb C1P5 was used as primary antibody to detect E6 protein in the tumor tissues, and the negative control was incubated with 18B7 mAb instead of primary antibody under the same conditions. Other reagents used in IHC were supplied with the kit.

### Western blot detection of HBx antigen in Hep 3B2.1-7 tumors

The Hep 3B2.1-7 tumors were homogenized on ice and western blot was performed as described above.

### Biodistribution and scintigraphic imaging with ^188^Re-labeled mAbs

For biodistribution and scintigraphic imaging in CasKi or Hep 3B2.1-7 tumor-bearing mice, mice were divided into groups of three and injected IP with either 100 µCi of specific mAbs ^188^Re-C1P5 or ^188^Re-4H9 or with control mAb ^188^Re-18B7. Twenty-four hours post-injection mice were anesthetized with Isoflurane and scintigraphically imaged for 2 min on a Siemens gamma camera equipped with ICON image processing software. Forty eight hours post-injection mice were sacrificed and the tumors and muscle were removed, blotted from blood, weighted, counted in a gamma counter and percentage of injected dose per gram tissue and tumor to muscle ratios were calculated.

### Therapy of CaSki and Hep 3B2.1-7 tumors in nude mice with ^188^Re-labeled mAbs

For therapeutic studies mice with tumors of 0.3–0.7 cm in diameter were randomized into the groups of six. For CasKi tumor-bearing mice, group # 1 was treated IP with 350 µCi ^188^Re-C1P5 mAb, group # 2–with matching amount (30 µg) “cold” C1P5 mAb, and group # 3 was left untreated. For RIT of Hep 3B2.1-7 tumor-bearing mice, group # 1 was given IP 240 µCi ^188^Re-4H9 mAb, group # 2–280 µCi, # 3–350 µCi, # 4–500 µCi, # 5–600 µCi of ^188^Re-4H9 mAb, # 6–30 µg “cold” 4H9 mAb and group # 7 was left untreated. Mice were observed for their well–being and tumor growth. The size of the tumor was measured every 3 days with calipers in three dimensions and the tumor volume was calculated as a product of three dimensions divided by 2. For histological analysis the tumors were removed from the mice at the completion of the experiments, fixed in buffered formalin, parafinized, cut into 5 µm slices and stained with hematoxylin and eosin (H&E).

### Statistical analysis

Non-parametric Wilcoxon Rank Sum test was used to compare organs uptake in biodistribution studies and tumor sizes in therapy studies. The differences were considered statistically significant when P values were<0.05.
